# Chromium-Containing Traditional Chinese Medicine, Tianmai Xiaoke Tablet, for Newly Diagnosed Type 2 Diabetes Mellitus: A Meta-Analysis and Systematic Review of Randomized Clinical Trials

**DOI:** 10.1155/2018/3708637

**Published:** 2018-03-07

**Authors:** Yuming Gu, Xuemin Xu, Zhe Wang, Yunsheng Xu, Xiuzhi Liu, Lejun Cao, Xueyang Wang, Zhengxin Li, Bo Feng

**Affiliations:** ^1^Shandong University of Traditional Chinese Medicine, Jinan 250355, China; ^2^Affiliated Hospital of Shandong University of Traditional Chinese Medicine, Jinan 250011, China; ^3^Second Affiliated Hospital of Shandong University of Traditional Chinese Medicine, Jinan 250001, China; ^4^Jinan Municipal Hospital of Traditional Chinese Medicine, Jinan 250012, China; ^5^Shanghai University of Traditional Chinese Medicine, Shanghai 201203, China; ^6^Shandong Province Qianfoshan Hospital, Shandong University, Jinan 250014, China

## Abstract

**Objective:**

Chromium-containing traditional Chinese medicine Tianmai Xiaoke tablet (TMXKT) is approved for treating newly diagnosed type 2 diabetes mellitus (T2DM) in China. This review aimed to compile the evidence from randomized clinical trials (RCTs) and quantify the effects of TMXKT on newly diagnosed T2DM.

**Methods:**

Seven online databases were investigated up to March 20, 2017. The meta-analysis included RCTs investigating the treatment of newly diagnosed T2DM, in which TMXKT combined with conventional therapy was compared with placebo or conventional therapy. The risk of bias was evaluated using the Cochrane Collaboration tool. The estimated mean difference (MD) and the standardized mean difference were within 95% confidence intervals (CI) with respect to the interstudy heterogeneity. The outcomes were measured using fasting blood glucose (FBG), 2-h postprandial blood glucose (2hPG), glycosylated hemoglobin A1c (HbA1c), and body mass index (BMI) levels.

**Results:**

TMXKT combined with conventional therapy lowered FBG level (MD = −0.68, 95% CI −0.90 to −0.45, *P* < 0.00001), 2hPG (MD = −1.33, 95% CI −1.86 to −0.79, *P* < 0.00001), HbA1c (MD = −0.46, 95% CI −0.57 to −0.36, *P* < 0.00001), and BMI (MD = −0.77, 95% CI −1.12 to −0.41, *P* < 0.00001).

**Conclusions:**

TMXKT combined with conventional therapy is beneficial for patients with newly diagnosed T2DM. However, the effectiveness and safety of TMXKT are uncertain because of the limited number of trials and low methodological quality. Therefore, practitioners should be cautious when applying TMXKT in daily practice. Also, well-designed clinical trials are needed in the future.

## 1. Introduction

Diabetes mellitus (DM) has now reached epidemic proportions globally. DM is characterized by hyperglycemia with disturbances in carbohydrate, fat, and lipid metabolism resulting from defects in insulin secretion, insulin action, or both [1]. The most recent data indicates 37.1 million patients with type 2 diabetes mellitus (T2DM) worldwide, of which 11.6% are from China. [2]. DM can lead to serious complications, including cardiovascular and kidney diseases, blindness, and amputations. It is a major cause of mortality. Hence, it is imperative that more attention is paid to the prevention and treatment of T2DM, especially newly diagnosed T2DM.

It is known that chromium deficiency leads to impaired glucose tolerance due to insulin resistance and hyperglycemia [3]. In China, chromium deficiency is common among patients with newly diagnosed with T2DM. Tianmai Xiaoke tablet (TMXKT; Hebei Fuge Pharmacy Co., Ltd, China) contains chromium picolinate (1.6 mg per tablet, equal to 200 *μ*g of chromium), Tianhuafen (Radix Trichosanthis, snakegourd root), Maidong (Radix Ophiopogonis, dwarf lilyturf tuber), and Wuweizi (Fructus Schisandrae Chinensis, Chinese magnolia vine fruit) in the ratio of 1.6 : 62.5 : 62.5 : 25. TMXKT is approved by the State Food and Drug Administration of China (state medical license number Z20049007) to treat T2DM and decrease glycated hemoglobin (HbA1c) levels in patients with diagnosed T2DM [4]. With the development of modern biological techniques, preclinical studies on the mechanism of TMXKT showed that the improvement in blood glucose occurred by activating the insulin-signaling pathway and inhibiting PTP1B and PCK2 in diabetic rats [5]. Furthermore, insulin resistance is alleviated through the PI3K/Akt pathway [6]. Further studies suggested that improvement in the blood glucose of diabetic rats involved increasing the expression of miR-375 and miR-30d to activate insulin synthesis in the islets [7]. In recent years, although a number of published clinical studies of TMXKT suggested that TMXKT was effective for treating newly diagnosed T2DM, a few systematic reviews have been published that summarized the effects of TMXKT. This systematic review and meta-analysis was performed to assess the strength of the current evidence to support the efficacy and safety of TMXKT for treating newly diagnosed T2DM.

## 2. Methods

This review protocol was registered with the International Prospective Register of Systematic Reviews (PROSPERO registration number CRD42017060132; available online at http://www.crd.york.ac.uk/PROSPERO/). It was written following the Preferred Reporting Items for Systematic Reviews and Meta-Analyses (PRISMA) reporting guidelines [8].

### 2.1. Database and Search Strategies

Seven databases, namely, PubMed, Embase, Cochrane Central Register of Controlled Trials (CENTRAL), China National Knowledge Infrastructure, Chinese Scientific Journal Database (VIP), Wanfang data, and Chinese Biomedical Literature Database (CBM), were investigated up to March 20, 2017. Because TMXKT is used mainly in China, a literature search was conducted in the four Chinese electronic databases to include the maximum possible number of clinical trials. The search was restricted to trials published in Chinese and English. The following search terms were used individually or in combination: “Tianmai Xiaoke Tablet,” “Tianmai Xiaoke pian,” “Tianmai Xiaoke tablet,” “Tianmai Xiaokepian,” “diabetes mellitus type 2,” “type 2 diabetes mellitus,” “type 2 diabetes,” “non-insulin-dependent diabetes mellitus,” “diabetes mellitus, stable,” and “diabetes mellitus, ketosis-resistant.” To increase the search range, no date and no language limits were imposed. Also, no restrictions on population characteristics were imposed. To include unpublished studies, the websites of the international clinical trial registry provided by the US National Institutes of Health (available at https://clinicaltrials.gov/) and the Chinese clinical trial registry (available at http://www.chictr.org.cn/index.aspx) were also searched. Furthermore, the reference lists of relevant retrieved studies were searched manually to identify any additional eligible studies. The authors of significant publications or experts in the relevant field were contacted for potential studies, and the pharmaceutical companies that manufactured TMXKT were also contacted to identify further published and unpublished studies. Two reviewers (Yuming Gu and Zhe Wang) independently screened the titles and abstracts for eligibility and examined the full text of the articles. Any discrepancies were resolved by consensus or after consulting a third party (Bo Feng).

### 2.2. Study Selection

All included trials met the following selection criteria: (1) the study was a randomized controlled trial (RCT); (2) the study examined patients with newly diagnosed T2DM, who received TMXKT combined with conventional therapy as treatment compared with those receiving placebo or conventional therapy alone; and (3) the study included participants irrespective of gender, age, or ethnicity, who were newly diagnosed with T2DM using clearly defined or internationally recognized criteria. The exclusion criteria were as follows: non-RCTs and quasi-RCTs.

### 2.3. Data Extraction

Two reviewers (Yuming Gu and Xuemin Xu) independently extracted data using a predesigned collection form. The following data were extracted: general trial characteristics (title, authors, and year); baseline patient and disease data (sample size, age, and gender); interventions (dose, details of control interventions); and outcomes (outcome measures, adverse events). Discrepancies were settled by consensus or a third party (Yunsheng Xu and Xiuzhi Liu).

### 2.4. Quality Assessment

Two reviewers (Xueyang Wang and Zhengxin Li) independently assessed the methodological quality of the RCTs using the Cochrane Collaboration Risk of Bias tool. The risk of bias was assessed according to the Cochrane Handbook [9], which consisted of six items: random sequence generation; allocation concealment; blinding of participants and personnel; blinding of outcome assessment; incomplete outcome data; and selective reporting and other sources of bias. Each item was categorized as “high risk” (at least one item had a high risk of bias), “low risk” (all items had a low risk of bias), or “unclear” (at least one item had an unclear risk of bias). Other biases included the sample calculation and profit bias.

### 2.5. Types of Outcomes

The treatment and control groups were compared in terms of their efficacy on fasting blood glucose (FBG), 2-h postprandial blood glucose (2hPG), glycosylated hemoglobin A1c (HbA1c), and body mass index (BMI).

### 2.6. Statistical Analysis

Data were analyzed using Review Manager 5.3 software (Cochrane Collaboration, Oxford, UK). Given the characteristics of the extracted data in the review, continuous outcomes were expressed as mean difference (MD) with 95% confidence intervals (CIs). *I*^2^ statistics were used to assess heterogeneity. A fixed-effects (FE) model was used if no significant heterogeneity was found in the data (*I*^2^ < 50%), and a random-effects (RE) model was used if significant heterogeneity was found (*I*^2^ ≥ 50%). Sensitivity analysis was performed to assess the stability of conclusions. Where heterogeneity was detected, accepted methods were used to explore the statistical heterogeneity using clinical parameters such as treatment duration, sample size, publication year, diagnostic criteria, and publication language. Publication bias was analyzed by funnel plot analysis if sufficient studies (*n* ≥ 10) were found.

## 3. Results

### 3.1. Description of Included Trials

**Figure 1:**
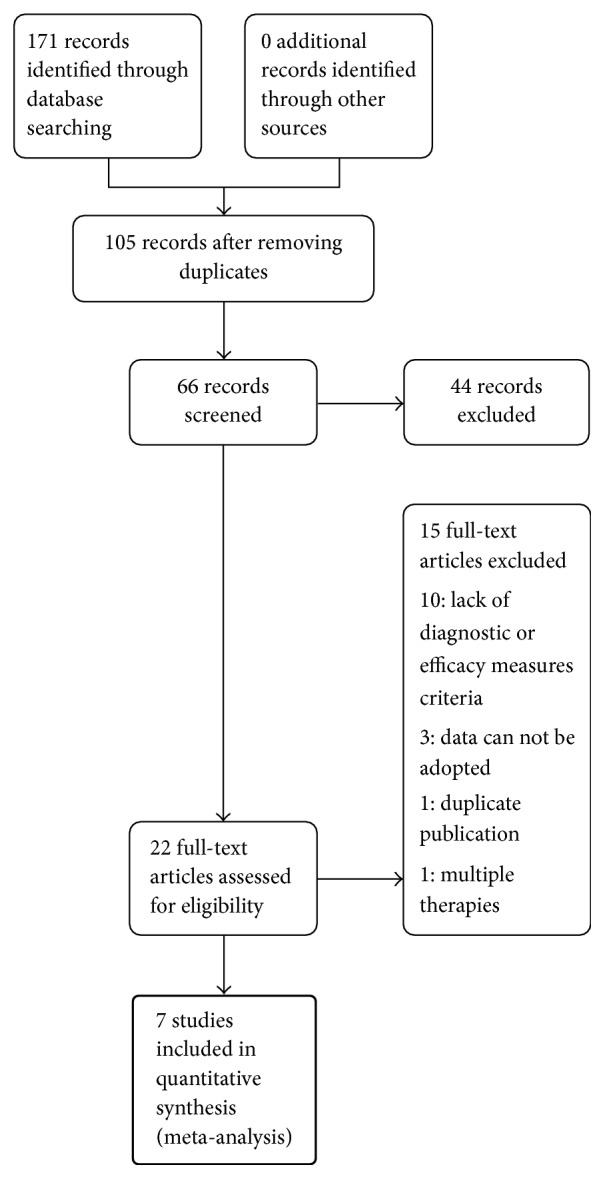
Flowchart of the trial selection process.

Among 171 identified studies, 7 studies were eligible for data extraction according to the inclusion and exclusion criteria [10–16]. The flow diagram for screening the trials is described in Figure 1. All studies were conducted in China. The language of the enrolled trials was Chinese.

### 3.2. Study Characteristics

The characteristics of the seven studies are described in Table 1. All the RCTs, including 360 patients from the treatment group and 357 controls, were included in this systematic review. In all the studies, participant characteristics were similar at baseline between different treatment groups. The trial duration ranged from 8 to 16 weeks: three studies lasted 12 weeks, two studies lasted 3 months, and the other two studies lasted 8 weeks and 16 weeks. Standard diagnostic diabetic criteria for newly diagnosed T2DM were applied to all included trials, including World Health Organization DM criteria (1999).

### 3.3. Risk of Bias in the Included Trials

**Figure 2:**
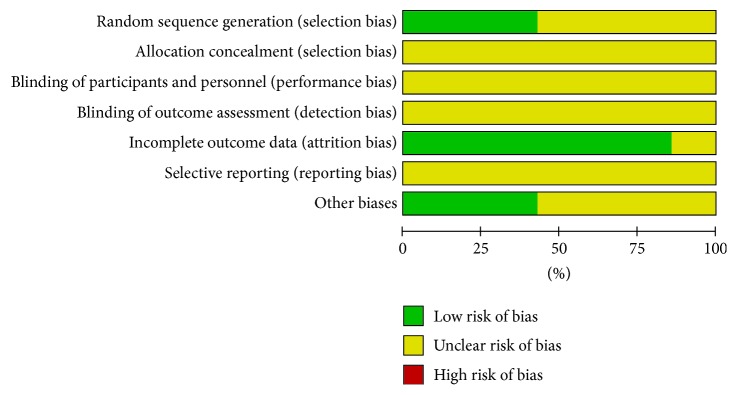
Risk of bias assessment in the included studies based on the Cochrane Handbook.

The quality and the risk of bias assessment of the included studies are described in Figure 2. Three studies used a random number table [10, 12, 15], and the other trials did not provide any detailed information regarding random sequence generation. Concealment of allocation, blinding of participants and researchers, and outcome assessment were achievable in a randomized, double-blind, and double-dummy controlled trial. However, all of the trials did not report concrete details on allocation concealment and blinding of outcome assessors. As a result, blinding of the participants and the researchers became difficult in this review. Incomplete outcome data were low risk in six studies [10–14, 16]. Selective reporting could not be judged in all the studies because of the insufficient information provided. Another bias was evaluated to be of low risk in all the studies.

### 3.4. Fasting Blood Glucose

**Figure 3:**
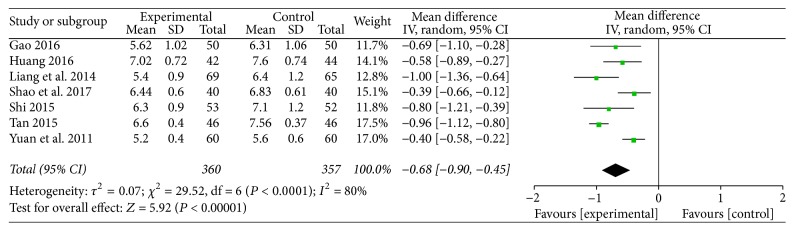
Random-effects meta-analysis of the effect of TMXKT combined with conventional therapy versus conventional therapy alone on the FBG level.

All studies investigated the effect of TMXKT on FBG level in newly diagnosed T2DM. The data were analyzed using an RE model according to the test of heterogeneity (*P* < 0.0001; *I*^2^ = 80%). All of the studies demonstrated a significant reduction in the FBG levels. For the principal outcome, MD for TMXKT combined with conventional therapy versus conventional therapy alone was −0.68 over the treatment period (95% CI −0.90 to −0.45; *P* < 0.00001). These data showed a significant difference in the FBG level between the two treatment groups (Figure 3).

### 3.5. Two-Hour Postprandial Blood Glucose

**Figure 4:**
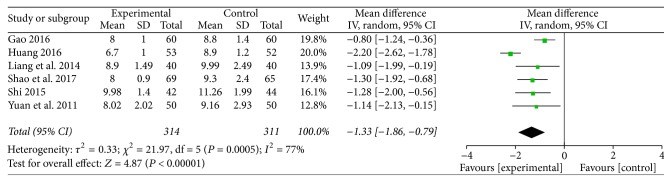
Random-effects meta-analysis of the effect of TMXKT combined with conventional therapy versus conventional therapy alone on the 2hPG level.

Six studies investigated the effect of TMXKT on the 2hPG level in newly diagnosed T2DM, providing an overall sample of 625 patients (314 in the experimental group and 311 in the control group) [10–15]. The data were analyzed using an RE model according to the test of heterogeneity (*P* = 0.0005; *I*^2^ = 77%). All the studies were reported as demonstrating a significant reduction in the 2hPG levels. For the principal outcome, MD for TMXKT combined with conventional therapy versus conventional therapy alone was −1.33 over the treatment period (95% CI −1.86 to −0.79; *P* < 0.00001). These data showed a significant difference in the 2hPG level between the two treatment groups (Figure 4).

### 3.6. Glycosylated Hemoglobin A1c

**Figure 5:**
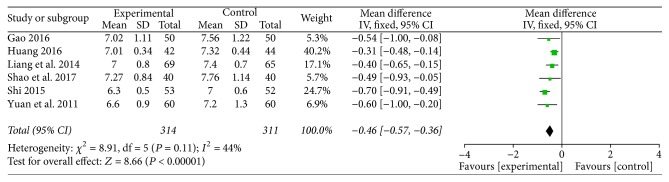
Fixed-effects meta-analysis of the effect of TMXKT combined with conventional therapy versus conventional therapy alone on the HbA1c level.

Six studies investigated the effect of TMXKT on the HbA1c level in newly diagnosed T2DM, providing an overall sample of 625 patients (314 in the experimental group and 311 in the control group) [10–15]. The data were analyzed using an FE model according to the test of heterogeneity (*P* = 0.11; *I*^2^ = 44%). All the studies demonstrated a significant reduction in the HbA1c level. For the principal outcome, MD for TMXKT combined with conventional therapy versus conventional therapy alone was −0.46 over the treatment period (95% CI −0.57 to −0.36; *P* < 0.00001). These data showed a significant difference in the HbA1c level between the two treatment groups (Figure 5).

### 3.7. Body Mass Index

**Figure 6:**
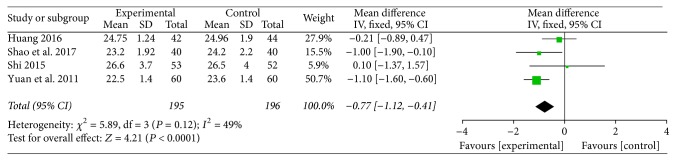
Fixed-effects meta-analysis of the effect of TMXKT combined with conventional therapy versus conventional therapy alone on the BMI level.

Four studies investigated the effect of TMXKT on the BMI level in newly diagnosed T2DM, providing an overall sample of 391 patients (195 in the experimental group and 196 in the control group) [11, 13–15]. The data were analyzed using an FE model according to the test of heterogeneity (*P* = 0.12; *I*^2^ = 49%). Three of the studies were reported as demonstrating a significant reduction in the BMI level [11, 13, 14]. For the principal outcome, MD for TMXKT combined with conventional therapy versus conventional therapy alone was −0.77 over the treatment period (95% CI −1.12 to −0.41; *P* < 0.00001). These data showed a significant difference in the BMI level between the two treatment groups (Figure 6).

### 3.8. Adverse Events

Adverse effects were reported in all studies. Common adverse events (AEs) occurring in the TMXKT group were gastrointestinal symptoms (nausea/vomiting, bloating, and diarrhea), nervous system symptoms, and hypoglycemia. No significant abnormality was seen in the routine blood examination or in the liver and renal function in all the studies.

### 3.9. Sensitivity Analyses

Post hoc sensitivity analysis was performed by limiting the meta-analysis to five trials [10, 12–15]. In these five trials, a few differences (or much overlap in the CIs) were found in the overall risk ratios of 2hPG. All *I*^2^ values were 0%, indicating low heterogeneity.

### 3.10. Publication Bias

Different intervention and outcome measurements indicated that funnel plot analysis could not be completed because of the small number of studies (<10) included in the meta-analysis.

## 4. Discussion

T2DM is a serious, yet manageable, medical condition that threatens public health. Nearly 10% of the world's population is affected by T2DM [6]. Therefore, prevention of T2DM is an important issue for medical researchers all over the world. As an adjunctive treatment method for T2DM, Chinese herbal medicines have been used in clinical practice for many years [17–19]. Two studies also indicated that plasma chromium concentrations were inversely associated with T2DM and pre-DM in Chinese adults [20, 21]. Relevant systematic reviews have shown that chromium can reduce FBG [22]. Chromium-enriched yeast compound was found to promote lipid metabolism in diabetic mice, elevate antioxidant capacity, and alleviate the symptoms of diabetes [23]. Chromium-enriched yeast was also found to significantly reduce blood glucose levels in diabetic rats and improve mesenteric microcirculation [24]. In addition, chromium chloride solution (CrCl3) decreased blood glucose levels in diabetic mice and improved the antioxidant activity [25]. The chromium-containing TMXKT also comprised three Chinese herbs: Tianhuafen, Maidong, and Wuweizi. A large number of studies have confirmed that these three kinds of traditional Chinese herbs include ingredients that can produce a hypoglycemic effect [24–29]. This review compared the effectiveness and safety of TMXKT combined with conventional treatment against conventional treatment alone for managing newly diagnosed T2DM. So far, the efficacy of TMXKT has not been evaluated systematically and comprehensively based on current international standards. This novel systematic review of the English and Chinese literature explored the efficacy and safety of TMXKT for newly diagnosed T2DM by integrating different outcome measures from seven RCTs.

In conclusion, this study demonstrated that TMXKT combined with metformin or pioglitazone or aspart insulin 30 had a more significant effect associated with the FBG level. The meta-analysis of the six trials presented significant reductions -in HbA1c and 2hPG levels when TMXKT combined with conventional treatment was used. The meta-analysis of four RCTs presented significant reductions in BMI when TMXKT combined with conventional treatment was used. However, the confirmation of the efficacy was limited due to poor methodological quality, insufficient placebo-controlled trials, and significant heterogeneity in the included trials. None of the RCTs included a placebo. Therefore, the effect of TMXKT was likely to be attributed to a placebo effect or other psychological effects. In addition, the reported AEs were not severe and required no additional special treatment, the most common being gastrointestinal symptoms.

### 4.1. Limitations

The RCTs included in the present meta-analysis had some limitations. All included studies declared randomization, but only three studies described a concrete random method [10, 12, 15]. Moreover, no trial described the allocation concealment method; inadequate allocation concealment might have created a potential selection bias and exaggerated any estimates of therapeutic effects. Additionally, most of the included trials were not registered, and also not long enough to evaluate the long-term effects of TMXKT. For sensitivity analyses, the outcomes of “FBG” and “2hPG” had high heterogeneity. Which study led to heterogeneity for “FBG” could not be found. For “2hPG,” one study [11] led to heterogeneity. Hence, it was believed that the high heterogeneity across studies was owing mainly to the use of different medications and the lack of high-quality literature.

Hence, this systematic review and meta-analysis was limited by the trials identified. Pooled analyses could not be performed due to high heterogeneity. Additionally, no clear description of dropouts and withdrawals was provided. The long-term effects of TMXKT were unknown, and no data were available regarding the improvement in diabetes after treatment. More trials with a high methodological quality and adequate power are needed to further identify the effectiveness and safety of TMXKT combined with conventional treatments. Also, the quality of life and long-term effect should be assessed.

### 4.2. Implications for Further Research

Although the present meta-analysis supported the effectiveness of TMXKT, double-blinded, placebo-controlled RCTs are necessary to validate the findings. Which ingredient of TMXKT is vital in treating newly diagnosed T2DM is worthy of further study. Moreover, a longer follow-up with TMXKT should be performed to assess its long-term effects on diabetes and the progression to diabetic complications.

## 5. Conclusions

Overall, TMXKT combined with conventional Western medicine is beneficial for improving FBG, 2hPG, HbA1c, and BMI levels for newly diagnosed T2DM.

Future studies should overcome the limitations to more precisely assess the effectiveness and safety of TMXKT.

## Figures and Tables

**Table 1:** Basic characteristics of the included studies.

First author, year	Sample size (T/C)	Population characteristics	Treatment	Control	Duration of treatment	Follow-up	Outcome assessment
Gao, 2016 [10]	100 (50/50)	T: mean age (51.32 years) M/F (28/22 cases) C: mean age (51.16 years) M/F (24/26 cases)	TMXKT 0.24 g/time, Bid + control	Metformin 0.5 g/time, Tid	3 months	None	FPG 2HPG HbA1c

Huang, 2016 [11]	86 (42/44)	T: mean age (NA) M/F (18/24 cases) C: mean age (NA) M/F (21/23 cases)	TMXKT 0.24 g/time, Bid + control	Metformin 0.5 g/time, Tid	12 weeks	None	FPG 2HPG HbA1c BMI

Liang, 2014 [12]	134 (69/65)	T: mean age (52 years) M/F (32/37 cases) C: mean age (56 years) M/F (29/36 cases)	TMXKT 0.24 g/time, Bid + control	Metformin 0.5 g/time, Tid	12 weeks	None	FPG 2HPG HbA1c

Shao, 2017 [13]	80 (40/40)	T: mean age (NA) M/F (NA) C: mean age (NA) M/F (NA)	TMXKT 0.24 g/time, Bid + control	Metformin 0.5 g/time, Bid	12 weeks	None	FPG 2HPG HbA1c

Yuan, 2011 [14]	120 (60/60)	T: mean age (NA) M/F (36/24 cases) C: mean age (NA) M/F (34/26 cases)	TMXKT 0.24 g/time, Bid + Metformin 0.25 g/time, Tid	Metformin 0.5 g/time, Tid	3 months	None	FPG 2HPG HbA1c BMI

Shi, 2015 [15]	105 (53/52)	T: mean age (NA) M/F (36/24 cases) C: mean age (NA) M/F (34/26 cases)	TMXKT 0.24 g/time, Bid + control	Aspart insulin 30 12–16 U/time, Bid	16 weeks	None	FPG 2HPG HbA1c BMI

Tan, 2015 [16]	92 (46/46)	T: mean age (50.18 years) M/F (33/13 cases) C: mean age (49.82 years) M/F (34/12 cases)	TMXKT 0.24 g/time, Bid + control	Pioglitazone, 15 mg/time Bid	8 weeks	None	FPG

BMI, body mass index; FPG, fasting blood glucose; HbA1c, glycosylated hemoglobin A1c; 2HPG, 2-h postprandial blood glucose; TMXKT, Tianmai Xiaoke tablet.
